# Multiple pulmonary cavities as the prominent manifestation of diffuse large B-cell lymphoma: a case report

**DOI:** 10.3389/fonc.2026.1752125

**Published:** 2026-04-21

**Authors:** Lili Zuo, Yilan Xia, Ruyi Zhang, Min Liu, Jianhui Fan, Lu Qian, Xiao Liang, An Xiao, Jiawei Geng

**Affiliations:** 1 Department of Infectious Diseases and Hepatic Diseases, The First People’s Hospital of Yunnan Province, The Affiliated Hospital of Kunming University of Science and Technology, Kunming, Yunnan, China; 2 Radiology Department, The First People’s Hospital of Yunnan Province, The Affiliated Hospital of Kunming University of Science and Technology, Kunming, Yunnan, China; 3 Department of Pathology, The First People’s Hospital of Yunnan Province, The Affiliated Hospital of Kunming University of Science and Technology, Kunming, Yunnan, China

**Keywords:** DLBCL, fever of unknown origin, FUO, lymphoma, pulmonary cavity

## Abstract

A 60-year-old man complaining of fever, lacking in strength and with poor appetite for half month, was admitted to our Department of Infectious Diseases and Hepatic Diseases. The patient’s chest computed tomography revealed multiple cavities in the lungs. And he was diagnosed with diffuse large B‐cell lymphoma following the positron emission tomography–computed tomography scan and laparoscopic lymph node biopsy. His symptoms and the pulmonary cavity lesions improved significant after eight cycles of rituximab, cyclophosphamide, doxorubicin, vincristine, and prednisone chemotherapy.

## Introduction

Diffuse large B-cell lymphoma (DLBCL) is the most common type of adult non-Hodgkin’s lymphoma, which may be found at various extranodal sites. The simultaneous involvement of lung, spleen, gastrointestinal tract, and lymph nodes in patients with DLBCL is uncommon. Pulmonary involvement is mostly reported in the form of lung nodules, mass, or pleural effusion. However, pulmonary involvement by DLBCL manifesting with pulmonary cavity is rarely reported. We reported an uncommon case of DLBCL presented with prominent multiple pulmonary cavities. Therefore, DLBCL should be considered as a differential diagnosis of primary lung cancer, infectious diseases, and metastatic lesions in patients with pulmonary cavity.

## Case report

A 60‐year‐old male patient presented to our emergency department complaining of fever, lacking in strength and with poor appetite for half month. The patient was admitted to our Department of Infectious Diseases and Hepatic Diseases on 17 July 2020 because of fever and whose chest computed tomography (CT) revealed multiple cavity-like lesions in the lungs. Routine anti-infective treatments at other hospitals had failed (details of the empirical anti−infective treatment were not accessible). He also had type 2 diabetes, with poor control of serum glucose due to irregular use of metformin tablets. He smoked half a pack of cigarettes daily for almost 40 years. Physical examination revealed poor general condition and emaciation, with a body mass index of 18.9 kg/m^2^. No other obvious abnormalities were observed.

**Figure 1:**
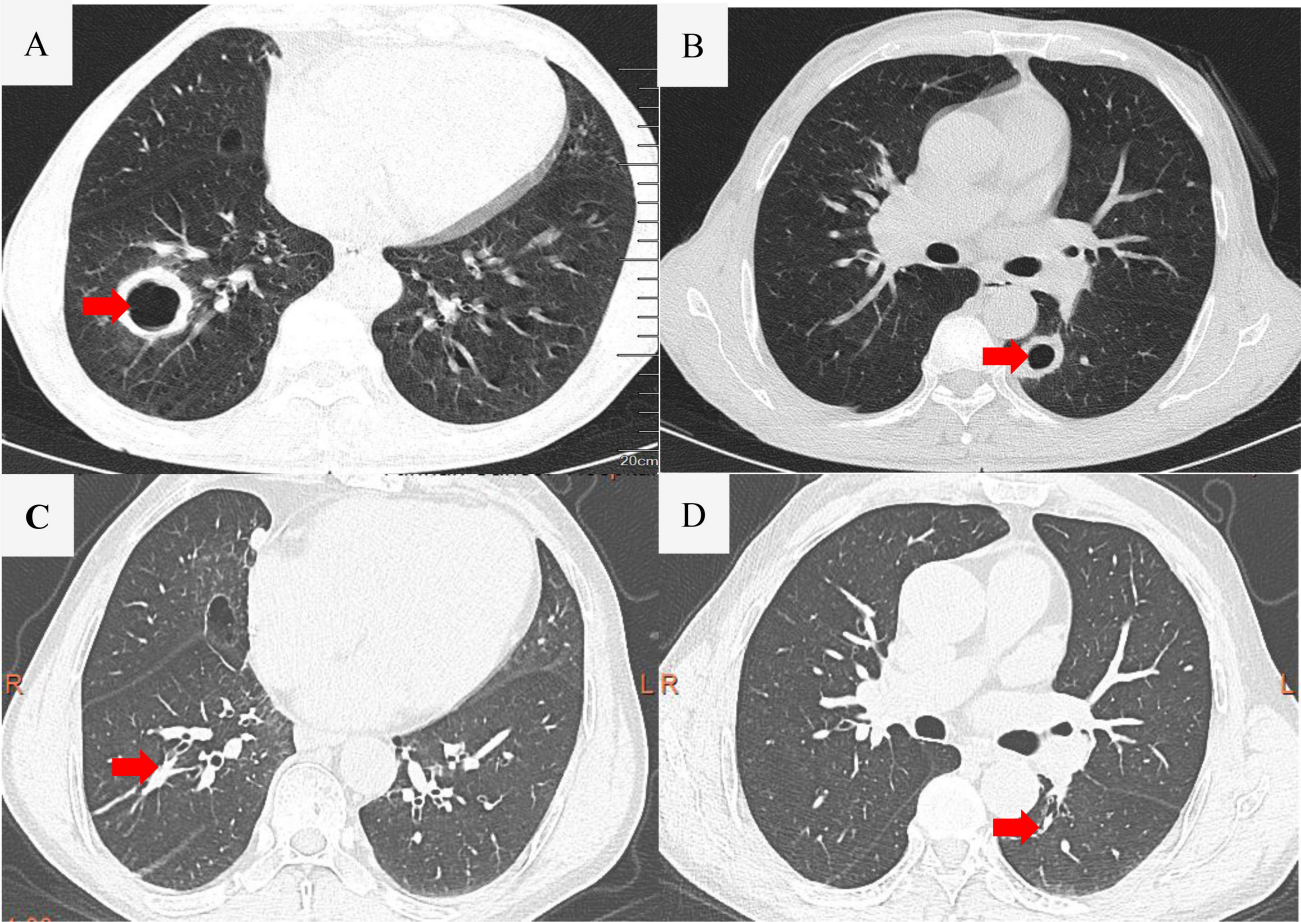
Images of chest computed tomography. The arrows in **(A, B)** indicate the affected areas. panel **(C, D)** show the absorption of lesions following treatment.

**Figure 2:**
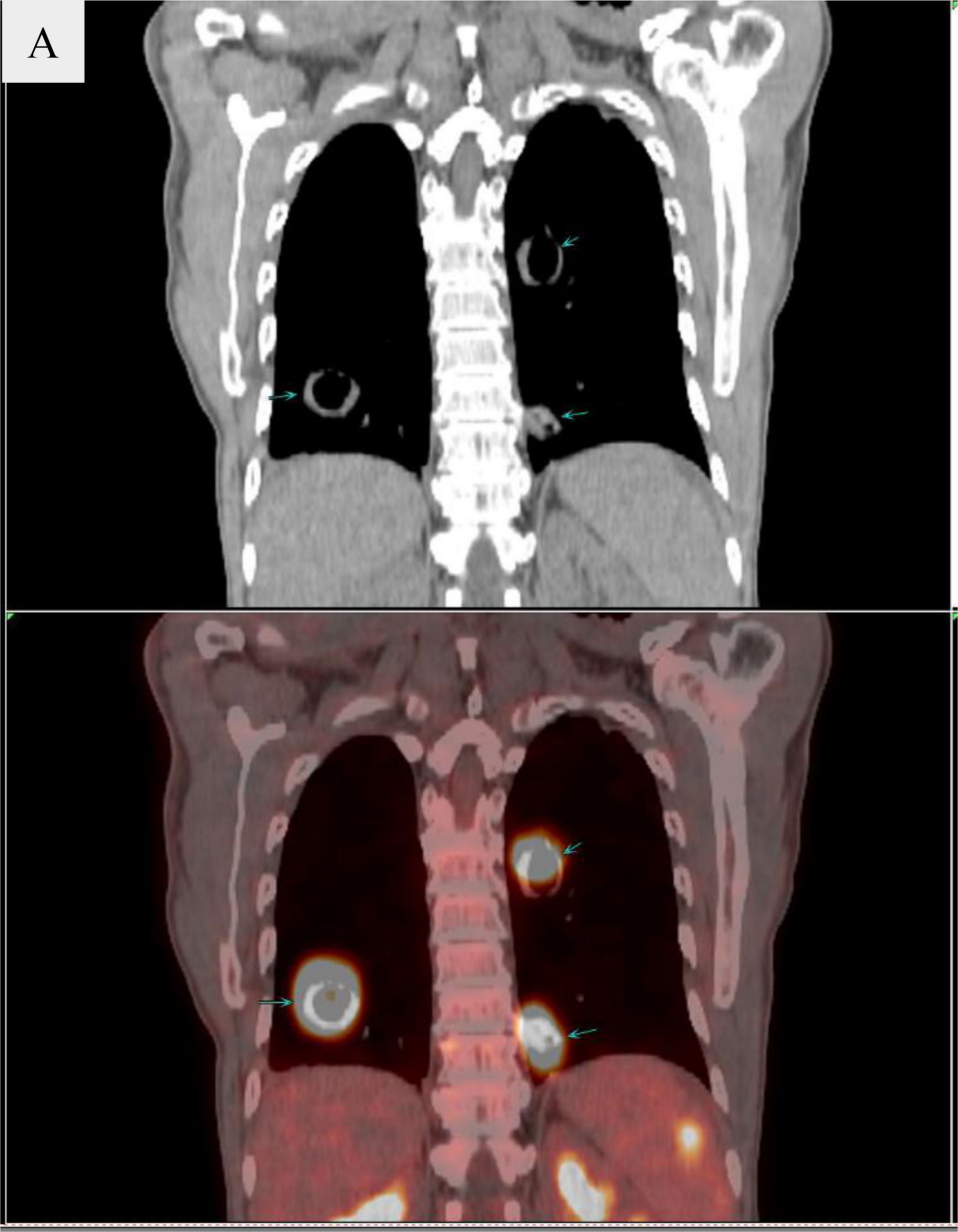
Images of PET‐CT. **(A)** shows High metabolism of the multiple thick-walled pulmonary cavities.

Laboratory studies showed C‐reactive protein level of 95.8 mg/L (reference range: 0–8), erythrocyte sedimentation rate level of 48 mm/h (reference range: 0–15), serum procalcitonin level of 0.30 ng/ml (reference range: 0–0.05), serum albumin level of 24.9 g/L (reference range: 35–55), serum lactate dehydrogenase level of 900 U/L (reference range: 120–250 U/L), serum ferritin level of 2,482.62 ng/ml (reference range: 21.81–274.86), serum glucose level of 7.9 mmol/L (reference range: 3.9–6.1), serum glycosylated hemoglobin level of 11.61% (reference range: 4.8–6.0%), serum sodium level of 131 mmol/L (reference range: 137–147 mmol/L), serum chloride level of 98 mmol/L (reference range: 99–110) and serum D-dimer level of 7.10 µg/ml (reference range: 0–1). No abnormality of the complete blood count, serum liver, and kidney function tests, and 1,3-β-D Glucan test were observed. Tumor marker tests showed neuron-specific enolase level of 25.06 ng/ml (reference range: 0–16.3) and carbohydrate antigen 125 level of 65.00 U/ml (reference range: 0–35). Interferon gamma release tests, repetitive blood cultures were negative. The DNA detection of Epstein-Barr virus in the peripheral blood was also negative. Screening for viral infections was performed, and serologic tests for hepatitis B virus, hepatitis C virus, and human immunodeficiency virus were all negative. The 18 items antinuclear antibody (ANA) panel and the test for the antineutrophil cytoplasmic antibody (ANCA) were negative. The chest CT demonstrated that multiple thick walled cavitary lesions were scattered in both lungs (**Figure 1**). The results of the bronchoscopy were almost normal. The bronchoalveolar lavage fluid (BALF) cultures for bacteria, fungi, mycobacteria, and nocardia were all negative. The abdomen contrast-enhanced CT showed obvious enhancement in the horizontal segment of duodenum and the group 6 intestinal wall, spleen and multiple enlarged lymph nodes near the retroperitoneal vessels with a diameter of about 3.2 cm for the larger ones in which necrosis was observed. The subsequent positron emission tomography -computed tomography (PET‐CT) scan was performed because of multisystem lesions and the need to exclude malignant diseases. And it demonstrated an elevated standard uptake value (SUV), ranging from 10.1 to 18 for the multiple thick-walled pulmonary cavities, enlarged cervical axillary, mediastinal, retroperitoneal and paravertebral lymphadenopathy, multiple splenic masses and segmental wall enlargement in small intestine of Group 6 and horizontal segment of duodenum (**Figure 2**). Meanwhile thick-walled cavity lesions were scattered in the both lung with significantly increased metabolisms, which should be considered lung infiltration of lymphoma, and other special infections should be excluded.

**Figure 3:**
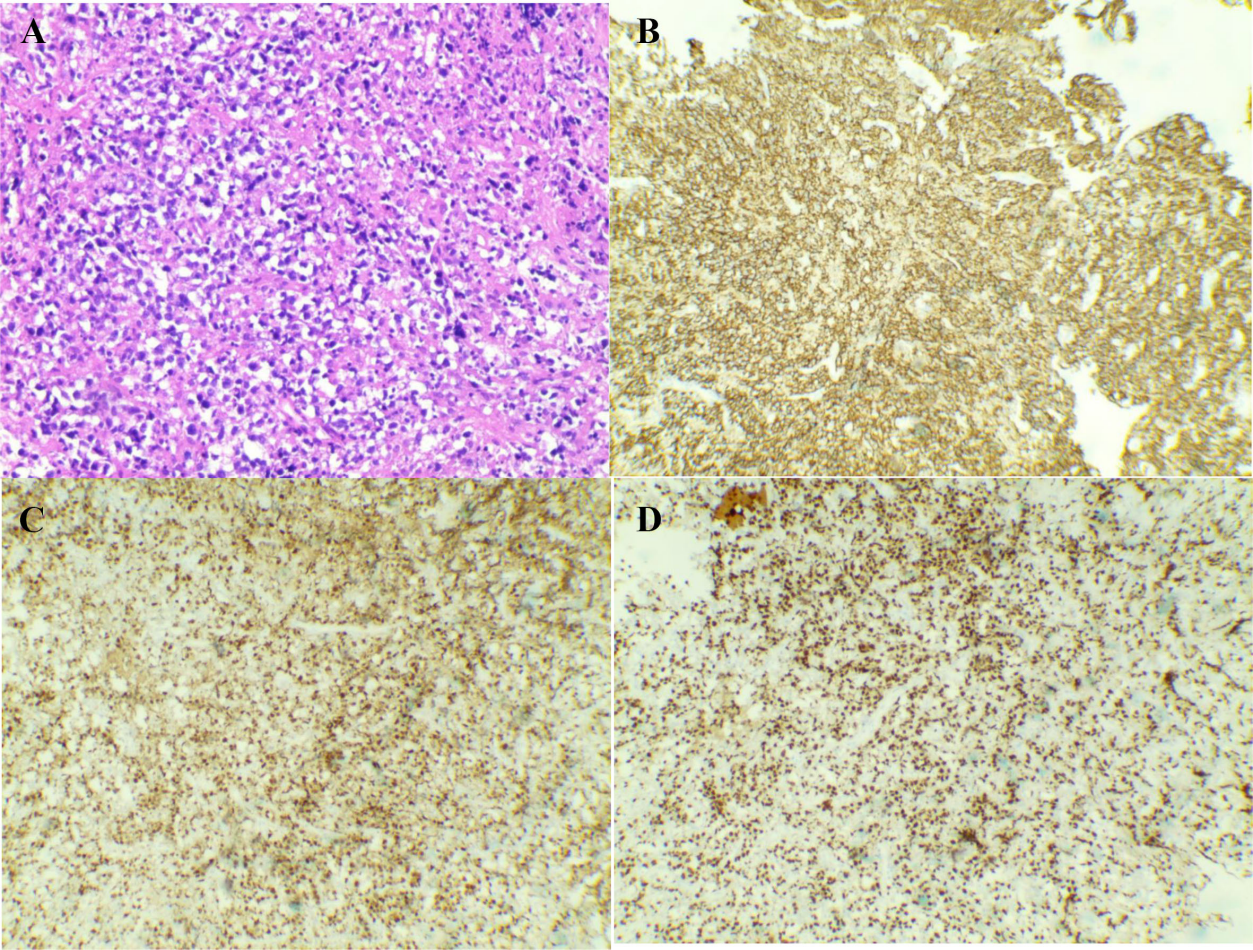
Pathological images of lymph node biopsy. **(A)** shows haematoxylin and eosin (HE) staining, revealing disruption to the normal architecture of the lymph node, with lymphocytic hyperplasia and necrosis. **(B)** shows CD20 membrane positivity in tumour cells. **(C)** demonstrates MUM-1 nuclear positivity in tumour cells. **(D)** shows PAX-5 nuclear positivity in tumour cells. All images are magnified at 100×.

Although the patient presented with multiple pulmonary cavities, consultation with the interventional radiology team indicated that percutaneous biopsy of these cavitary lesions carried a high procedural risk and was therefore not recommended as the initial diagnostic approach. In contrast, abdominal CT and PET−CT revealed multiple enlarged, hypermetabolic lymph nodes in the retroperitoneal region. Considering both safety and diagnostic yield, we selected laparoscopic retroperitoneal lymph node biopsy as the preferred method for obtaining tissue for pathological confirmation. Excision biopsy of retroperitoneal lymph nodes was suggestive of DLBCL non-GCB type, which was confirmed by bright CD20, LAC, PAX-5 and MUM1 positivity on immunohistochemistry (**Figure 3**). The International Prognostic Index (IPI) score was 5, and the Central Nervous System International Prognostic Index (CNS-IPI) score was 4. Empirical treatment with Tazobactam and Piperacillin had no effect. When the diagnosis of DLBCL was made, the patient had received eight cycles of rituximab, cyclophosphamide, doxorubicin, vincristine, and prednisone (R-CHOP) chemotherapy in the hematology department of Yunnan cancer hospital. The general condition of the patient had improved and the symptoms of fatigue, anorexia, cough and fever had improved under the treatment. The chest and abdominal CT reexamined by Yunnan cancer hospital from September 2020 to January 2021 showed that the multiple pulmonary cavity lesions had almost disappeared (**Figure 1**), and the multiple lymph nodes behind the upper, middle, and lower abdominal membranes and the splenic nodules were smaller than before.

A telephone follow−up in December 2021 indicated that the patient’s general condition was stable. However, due to financial limitations, the patient did not undergo regular scheduled imaging follow−up. The patient experienced disease relapse in 2023, responded poorly to subsequent treatment, and passed away in November 2023. The interval from completion of therapy to the last follow−up was approximately 34 months.

## Discussion

A variety of adult lung diseases can be manifested as pulmonary cavity disease. Based on the underlying etiology of cavity formation, pulmonary cavities are generally classified into infectious and non−infectious types. At present, reported infectious cavitary lung diseases include lung abscesses caused by common bacteria such as *Staphylococcus aureus* and *Klebsiella pneumoniae*, as well as pulmonary cavities resulting from special pathogenic microorganisms such as fungi, *Mycobacterium tuberculosis*, and Paragonimus. In contrast, non-infectious cavitary lung diseases comprise primary lung neoplasms, metastatic tumors, Wegener’s granulomatosis (1), and immunoglobulin G4 related lung disease (2). It is well known that cavities are divided into thin-walled cavities and thick-walled cavities. Different types of pulmonary cavity may tend to consider different diseases. Still some signs overlap and confuse each other on the image, which is easy to lead to misdiagnosis and mistreatment. In this patient, infectious cavitation was initially suspected given the clinical context of an elderly male with fever, elevated infection markers, a history of diabetes, and poor glycemic control. Chest CT revealed multiple thick−walled cavities characterized by relatively uniform wall thickening, non−eccentricity, rough margins, and surrounding patchy exudation. Notably, there were no features of lobulation, spiculation, or halo sign. The inner wall appeared smooth, without evidence of hyperplastic nodules, filamentous organisms, crescent sign, or air−fluid levels, and there were no signs of bronchial inflation. However, no evidence of tuberculosis, fungal infection, or other pathogens was identified, as blood tests, sputum cultures, and bronchoalveolar lavage all yielded negative results. Moreover, routine anti-infective therapy was ineffective. After a series of examinations, especially PET-CT and laparoscopic lymph node biopsy, the final diagnosis of DLBCL non-GCB type was made according to lymph node examination and immunohistochemistry, and he was determined as IVB stage from extranodal lesions and systemic symptoms. At present, R-CHOP remains the standard treatment for DLBCL, which significantly improves the long-term survival rate of patients (3, 4), and it makes DLBCL to be a curable malignant tumor. After eight cycles of treatment with R-CHOP chemotherapy, the imaging findings showed that the multiple pulmonary cavitary lesions were basically absorbed, which also confirmed that the pulmonary cavitary lesions were caused by lymphoma infiltration. Because the diagnosis was clear, the patient could receive timely and effective treatment. At present, the clinical symptoms and lesions of the patient are well controlled, which greatly improves the survival rate and quality of life of the patient.

DLBCL has a variety of clinical manifestations, including painless progressive lymphadenopathy, fever, hepatosplenomegaly, and various forms of extranodal infiltration. However, the onset and progress of the disease is hidden, which may delay patient recognition and medical attention. Reported pulmonary involvement mainly in lymphoma has include primary pulmonary DLBCL manifested as lung nodules and masses (5), pulmonary mucosa associated lymphoid tissue lymphoma (6), DLBCL with lung consolidation (7), DLBCL with double lung ground glass shadow (8), DLBCL with multiple ground-glass nodules (9) and DLBCL with lung cavity (10) (11). Pulmonary involvement in DLBCL has typically been reported as nodular lesions associated with cough, hemoptysis, and significant weight loss (12) (13). In contrast, our case was characterized by multiple cavitary lesions, representing an uncommon manifestation. This atypical presentation expands the recognized imaging spectrum of DLBCL and underscores the importance of considering lymphoma in the differential diagnosis of pulmonary cavities, particularly in diabetic patients.

In principle, patients with DLBCL who present with a high IPI score (>=4) are considered at increased risk for CNS involvement, and current guidelines generally recommend prophylactic intrathecal chemotherapy in such cases. Unfortunately, this patient received treatment at an outside institution, and a review of the medical records confirmed that intrathecal prophylaxis was not administered. This represents a deviation from standard practice and may have contributed to the patient’s subsequent disease course. The absence of central nervous system prophylaxis should therefore be acknowledged as a limitation in the management of this case.

In summary, DLBCL should be recognized as a potential cause of cavitary lung disease, and accurate diagnosis is essential to guide appropriate therapy and improve patient outcomes.

## Data Availability

The original contributions presented in the study are included in the article/supplementary material. Further inquiries can be directed to the corresponding authors.

## References

[1] Russell B Mohan S Chahal R Carette S Pagnoux C . Prognostic significance of cavitary lung nodules in granulomatosis with polyangiitis (Wegener’s): a clinical imaging study of 225 patients. Arthritis Care Res (Hoboken). (2018) 70:1082–9. doi: 10.1002/acr.23443. PMID: 28992397

[2] Xie LJ Li JF Liu Z Zhang F Zhao C Qin LP . Immunoglobulin G4-related lung disease presenting as lung cavitating mass and mimicking lung cancer. Arch Rheumatol. (2017) 32(4):365–369. doi: 10.5606/ArchRheumatol.2017.6337. PMID: 29901029 PMC5868464

[3] González-Barca E Coronado M Martín A Montalbán C Montes-Moreno S Panizo C . Spanish Lymphoma Group (GELTAMO) guidelines for the diagnosis, staging, treatment, and follow-up of diffuse large B-cell lymphoma. Oncotarget. (2018) 9(49):32383–32399. doi: 10.18632/oncotarget.25892. PMID: 30190794 PMC6122355

[4] Tilly H Vitolo U Walewski J Gomes da Silva M Shpilberg O André M . Diffuse large B-cell lymphoma (DLBCL): ESMO Clinical Practice Guidelines for diagnosis, treatment and follow-up. Ann Oncol. (2015) 26:v116–v125. doi: 10.1093/annonc/mds273. PMID: 22997459

[5] Zhu Z Liu W Mamlouk O O'Donnell JE Sen D Avezbakiyev B . Primary pulmonary diffuse large B cell non-Hodgkin’s lymphoma: a case report and literature review. Am J Case Rep. (2017) 18:286–290. doi: 10.12659/ajcr.901528. PMID: 28321110 PMC5373819

[6] Du C Zhang J Wei Y Bai J Duan MC Liu G . Retrospective analysis of 9 cases of primary pulmonary mucosa-associated lymphoid tissue lymphoma and literature review. Med Sci Monit Basic Res. (2018) 24:233–240. doi: 10.12659/msmbr.912762. PMID: 30581188 PMC6698091

[7] Jandial A Mishra K Dey P Goni D Umair M Lad D . Diffuse large B-cell lymphoma: a rare cause of lung consolidation. Indian J Hematol Blood Transfus. (2018) 34(4):768–771. doi: 10.1007/s12288-018-0993-2. PMID: 30369763 PMC6186225

[8] Inaty H Artiles C Yadav R Garcha P Mukhopadhyay S Sahoo D . Diffuse large B-cell lymphoma presenting as diffuse bilateral ground-glass opacities and diagnosed on transbronchial lung biopsy. Ann Am Thorac Soc. (2017) 14:605–7. doi: 10.1513/annalsats.201610-833le. PMID: 28362535

[9] Wang Q Yan H Wang R Li C Li W Xu Y . Primary pulmonary diffuse large B-cell lymphoma with multiple ground-glass nodules as the primary manifestation: a case report. Med (Baltimore). (2020) 99(51):e23501. doi: 10.1097/md.0000000000023501. PMID: 33327289 PMC7738136

[10] Yamane H Ohsawa M Shiote Y Umemura S Suwaki T Shirakawa A . Cavitary pulmonary involvement of diffuse large B-cell lymphoma transformed from extra nodal marginal zone B-cell lymphoma MALT type. Clin J Gastroenterol. (2011) 4(6):401–406. doi: 10.1007/s12328-011-0259-0. PMID: 26189744

[11] Hibino Y Imai R Jinta T . Diffuse large B-cell lymphoma presenting with cavitary lung disease. Respirol Case Rep. (2020) 8:e00584. doi: 10.1002/rcr2.584. PMID: 32405417 PMC7214784

[12] Natarajan P Ng KL Huan NC Chong AR Momin N Mohd Aminudin NH . Lung cavity and endobronchial mass: an uncommon initial presentation of Hodgkin’s lymphoma. Respirol Case Rep. (2025) 13(2):e70254. doi: 10.1002/rcr2.70254. PMID: 40535729 PMC12175016

[13] Chen HY Kuo YC Cheng WC Chen WC . A rare presentation of anaplastic large cell lymphoma as a cavitary pulmonary mass with hypercalcemia. Thorac Cancer. (2022) 13(16):2398–2400. doi: 10.1111/1759-7714.14571. PMID: 35811296 PMC9376169

